# Characterization of Unsaponifiable Matter in Unripe *Pistacia atlantica* Fruits: Evaluation of Antioxidant Activity and *α*‐Amylase Inhibitory Effect

**DOI:** 10.1155/sci5/8016775

**Published:** 2025-12-29

**Authors:** Mokhtar Benmohamed, Mohamed Harrat, Mohammed Messaoudi, Amar Djemoui, Ahmed Souadia, Barbara Sawicka, Ayomide Victor Atoki, Mohamed Yousif

**Affiliations:** ^1^ Laboratoire des Sciences Fondamentales, Université Amar Télidji, Laghouat 03000, Algeria; ^2^ Laboratory of Research on Bioactive Products and Biomass Valorization, Department of Chemistry, Higher Normal School of Kouba (ENS), P.O. Box 92 Vieux-Kouba, Algiers 16308, Algeria; ^3^ Laboratory of Organic Chemistry and Natural Substances, Faculty of Exact Sciences and Computer, University of Djelfa, P.O. Box 3117, Djelfa 17000, Algeria; ^4^ Laboratory of Physico-Chemistry of Materials and Environment, Faculty of Exact Sciences and Computer, University of Djelfa, P.O. Box 3117, Djelfa 17000, Algeria; ^5^ Department of Plant Production Technology and Commodities Science, University of Life Sciences in Lublin, Akademicka 15 Str., Lublin 20-950, Poland, up.lublin.pl; ^6^ Department of Biochemistry, Kampala International University, Ishaka, Uganda, kiu.ac.ug

**Keywords:** *α*-amylase inhibition, antioxidant activity, carotenoids, fruit ripening stage, *Pistacia atlantica*, sterols, tocopherols, unsaponifiable matter

## Abstract

This study provides a comprehensive characterization of the unsaponifiable matter (USM) in oils extracted from *Pistacia atlantica* fruits during prematurity phases (unripe and semiripe stages) from two distinct biogeographical regions in Algeria (Sougaa and Bousdraya). Notably, quantitative analysis revealed substantial variations in bioactive compound concentrations: tocopherols ranged from 3.10 to 17.88 mg *α*TE/g USM, sterols from 844.45 to 871.36 mg *β*S/g USM, and carotenoids from 15.22 to 20.76 mg *β*CE/g USM, demonstrating the significant phytochemical richness of early developmental stages. The USM components were quantified using validated spectrophotometric methods, with parallel evaluation of antioxidant and *α*‐amylase inhibitory activities. The most remarkable finding was the exceptional antioxidant potency of semiripe fruits from Bousdraya (BRF), showing IC_50_ = 0.49 mg/mL, representing one of the strongest antioxidant activities reported for *Pistacia* species unsaponifiable fractions. The unripe sample from Sougaa (SRC) exhibited the highest USM yield (10.45%) along with superior carotenoid and sterol concentrations. Furthermore, semiripe fruits from Sougaa (SRF) demonstrated the strongest *α*‐amylase inhibition (34.58 μmol acarbose Eq/g USM), highlighting their potential for diabetes management applications. Correlation analysis revealed a strong negative relationship between tocopherol content and antioxidant activity (*r* = −0.92), confirming their role as primary radical scavengers. The results provide crucial insights for developing functional ingredients for nutraceutical and pharmaceutical industries targeting oxidative stress and glycemic control. Initial stages of *Pistacia atlantica* fruit development represent an underappreciated source of valuable bioactive compounds. These results clearly indicate that an optimal harvesting strategy must consider both geographic origin and phenological stage, depending on the desired functional profile (e.g., semiripe BRF for strong antioxidant activity, immature SRC for maximum USM, and carotenoid/sterol yield). The key role in this activity was confirmed by a strong, statistically significant, negative correlation between tocopherol content and the IC_50_ value (*r* = −0.92). However, the highest alpha‐amylase inhibition activity, significantly different from the immature samples (*p* < 0.05), was achieved by semiripe samples from Sougaa (SRF, 34.58 p.m. 1.33 mµmol AACE/g USM) and BRF, suggesting that other, statistically uncorrelated (*r* approx. 0.01 for tocopherols) compounds are responsible for this effect. In terms of content, the immature sample from Sougaa (SRC) was statistically superior in terms of carotenoid and sterol content. These findings definitively confirm that the selection of harvest site and time is critical for maximizing the functional quality of the oil.

## 1. Introduction

Vegetable oils are among the essential biological sources of dietary fats, providing not only vital energy but also essential lipid compounds for health, particularly unsaturated fatty acids [1, 2]. These oils have garnered growing scientific interest not only for their saponifiable fatty acid content but also for their valuable minor constituents known as the unsaponifiable matter (USM) [3]. This fraction encompasses a wide array of biologically active compounds, the most common of which include plant sterols, tocopherols, carotenoids, polyphenols, and squalene [4]. Although the USM typically constitutes less than 2% of the total oil mass [5], it plays a fundamental role in enhancing the nutritional and functional properties of vegetable oils. This is attributed to its diverse biological activities, such as antioxidants, cholesterol‐lowering, anti‐inflammatory, anticancer, immunomodulatory, antimicrobial, analgesic, and wound‐healing effects [6]. These properties make the unsaponifiable fraction a prime candidate for developing nutraceuticals and functional foods aimed at promoting health and preventing chronic diseases [51, 52].

Tocopherols are considered among the most potent natural antioxidants and serve as a primary source of vitamin E [7]. Meanwhile, plant sterols are known for their ability to inhibit cholesterol absorption in the intestine, thereby contributing to the reduction of low‐density lipoprotein (LDL) cholesterol and the prevention of cardiovascular diseases [8]. Carotenoids, on the other hand, play a crucial role as antioxidants and precursors of vitamin A, while also contributing to the sensory and color attributes of oils [9]. The proportion and composition of these unsaponifiable compounds are influenced by several factors, most notably the plant species, environmental growing conditions, extraction methods, and the phenological ripening stage of the fruits [6, 10, 11, 49].

In this context, *Pistacia atlantica* is one of the plant species widely distributed across the Mediterranean region [12, 50]. It is a fast‐growing species belonging to the Anacardiaceae family, which grows spontaneously in various soil types [13, 14] and is tolerant to a wide range of climatic conditions [15]. The tree can reach heights of up to 20 m and trunk diameters of up to 1 m. It typically grows either as isolated individuals or in dense groups, even in harsh environments where few other species can thrive [16]. The altitudinal range of *Pistacia atlantica* extends from one hundred meters above sea level to much higher elevations, such as three thousand meters in eastern Algeria [17]. This species comprises three recognized subspecies: *cabulica, kurdica*, and *mutica* [18]. Various parts of *Pistacia atlantica*, including the fruits, bark, and resin, have been used in traditional medicine for their therapeutic properties. These parts have been employed in the treatment of liver, kidney, and heart diseases and respiratory disorders and wound healing and for a variety of purposes as a tonic, antiseptic, and antihypertensive agent. Additionally, they have shown beneficial effects in managing gastrointestinal and digestive disorders [19].

Although numerous studies have investigated the chemical composition of oils derived from various parts of *Pistacia atlantica*, particularly from mature fruits [11, 20–24], data regarding the early stages of fruit development remain limited. This gap is significant, as the early ripening phase is a period of intense metabolic activity where the biosynthesis of many valuable secondary metabolites is at its peak, potentially leading to a unique and richer profile of bioactive unsaponifiable compounds compared to the mature stage. This is noteworthy given that this developmental phase is marked by significant physiological transformations, especially in the accumulation of secondary metabolites. Main study objective: to assess the effect of maturity stage (immature vs. semiripe) and geographical origin (Sougaa vs. Bousdraya) of *Pistacia atlantica* fruits on the concentration and profile of unsaponifiable bioactive compounds (tocopherols, sterols, and carotenoids) and their antioxidant and *α*‐amylase‐inhibiting activities, in order to identify optimal harvesting conditions for nutraceutical and pharmaceutical applications. Moreover, the study evaluates the biological activity of these compounds as antioxidants and natural inhibitors of *α*‐amylase, a key enzyme involved in starch degradation and blood glucose regulation. The novelty of this work lies in its focus on the often‐overlooked early developmental stages of the fruit, which may offer a superior and distinct source of these bioactive compounds for exploitation. The importance of this work lies in its contribution to unveiling the health‐promoting and functional potential of oil derived from early‐stage *Pistacia atlantica* fruits, thereby opening new perspectives for its utilization as a promising natural ingredient in health‐oriented food and pharmaceutical industries. The findings are expected to provide crucial data for optimizing harvest timing to maximize the nutraceutical value of the oil, guiding its potential application in the development of dietary supplements, functional foods for managing oxidative stress and diabetes, and natural pharmaceutical ingredients.

## 2. Materials and Methods

### 2.1. Sample Collection

**Figure 1:**
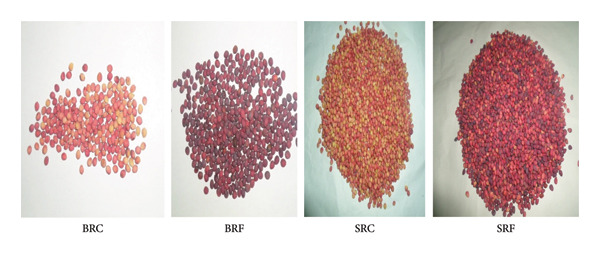
Samples of *Pistacia atlantica* fruits: unripe stage (SRC = Sougaa, unripe; SRF = Sougaa, middle maturity; BRC = Bousdraya, unripe; BRF = Bousdraya, middle maturity).

The samples of unripe fruit of *P. atlantica* were collected in August 2018 from two different parts of Djelfa Province, Algeria; the first region S is Sougaa Bouira, Al Hadab, which is symbolized by SRC, SRF (latitude: 35°10′59.9″N; longitude: 2°55′13.5″E, altitude: 856 m). The second region B is Bousdraya Ain Oussara (BRC, BRF) (latitude: 35°22′2.3″N; longitude: 2°56′27.0″E; altitude: 720 m). Figure 1 shows the image of the samples. The plant material was botanically identified and confirmed by Professor M. Yousfi, Laghouat University, Algeria. The seeds’ part samples were dried in the open air, in the shade, and away from sunlight and moisture for 4 weeks. The samples were ground to a fine powder using an agate mortar and pestle.

### 2.2. Oil Extraction

The oil was extracted following the standard protocol established by the Association of Official Analytical Chemists (AOAC, 2000) [25], using a Soxhlet apparatus and petroleum ether as the solvent. A precisely weighed amount of one hundred grams of each dried sample (for three replicates per sample) was used to conduct the extraction process, and the extraction was conducted for 4–6 h until the solvent became clear, indicating complete extraction. The obtained oil extract was then collected for the subsequent isolation of the USM. The oil yield was expressed as a percentage and calculated based on the weight of the oil obtained after solvent evaporation, using the following formula:

(1) crude extract content= mass of the extractmass of the test sample plant material ×10.

### 2.3. Unsaponifiable Fraction Preparation

The USM was isolated according to the method described in reference [26]. A weighed portion of the oil was dissolved in 100 mL of ethanolic potassium hydroxide solution (1 N KOH) and refluxed for 1 h with constant stirring. After cooling, 100 mL of distilled water was added, and the unsaponifiable fraction was extracted three times with 100 mL of diethyl ether using liquid–liquid extraction. The combined organic phases were dried with anhydrous Na_2_SO_4_ and filtered, and the solvent was evaporated at 40°C under reduced pressure. The yield of the USM was then calculated.

### 2.4. Phytochemical Analysis

#### 2.4.1. Tocopherol Determination

Total tocopherols were quantified spectrophotometrically following the method described in reference [27], which is based on the reduction of ferric ions (Fe^3+^) to ferrous ions (Fe^2+^) by tocopherols in the presence of 1,10‐phenanthroline, forming an orange‐colored complex measured at 510 nm. *α*‐Tocopherol was used as an external standard to generate a calibration curve within the concentration range of 0.012–0.062 mg/mL. Ethanol was used as the extraction solvent. Tocopherol concentrations were calculated using the linear regression equation from the calibration curve and expressed as mg/g of oil.

#### 2.4.2. Sterol Determination

Total sterols were determined using the Liebermann–Burchard spectrophotometric method as described in reference [28]. This method relies on a characteristic colorimetric reaction of 3*β*‐hydroxysteroids containing a double bond at the five to six position. In an acidic environment, sterols react with *β*‐acetic anhydride to form a stable complex that exhibits absorbance in the visible range at 550 nm. The Liebermann reagent used for this purpose consists of a mixture of 60 mL acetic anhydride, 10 mL concentrated sulfuric acid, and 30 mL acetic acid. For the assay, 1 mL of each diluted solution was combined with 2 mL of the Liebermann reagent and allowed to stand for 25 min to ensure full color development and stabilization. *β*‐Sitosterol, dissolved in chloroform, was used as the calibration standard over the concentration range of 0.012–0.062 mg/mL. The sterol content of the samples was quantified using a calibration curve and results were expressed as milligrams per gram of oil (mg/g).

#### 2.4.3. Carotenoid Determination

Total carotenoids were estimated according to the spectrophotometric method described in reference [29], based on the direct absorbance of *β*‐carotene at 460 nm in chloroform. A calibration curve was prepared using *β*‐carotene standards (0.012–0.060 mg/mL). The concentration of carotenoids in oil extracts was calculated using the slope of the standard curve and absorbance values, with results expressed as mg/g of oil.

In all quantifications, the following formula was used to calculate concentrations:

(2) Cmgg=AK×F×VP,

where *A*: absorbance, *K*: slope of the calibration curve, *F*: dilution factor, *V*: volume of solvent (3 mL), and *p*: mass of oil sample.

### 2.5. Biological Activity Assays

#### 2.5.1. DPPH Radical Scavenging Activity

The antioxidant activity of the unsaponifiable extracts was evaluated by [30]. In brief, 0.5 mL of the sample, previously diluted in absolute ethanol, was mixed with 0.5 mL of a 250 μM DPPH solution prepared in the same solvent. The reaction mixture was vigorously shaken and left to incubate in the dark at ambient temperature for 30 min. Subsequently, the absorbance was measured at 517 nm against a blank, to assess the sample’s free radical scavenging activity. The DPPH radical inhibition percentage (PI%) was determined using the following equation:

(3) PI%=A0−AA0∗100%,

where *A*_0_: absorbance without inhibitor and *A*: absorbance of the sample with inhibitor.

Dose–response curves were plotted (concentration vs. % inhibition), and IC_50_ values (concentration required to inhibit 50% of DPPH radicals) were determined for each extract.

#### 2.5.2. *α*‐Amylase Inhibitory Activity

The *α*‐amylase inhibitory activity was assessed using the colorimetric DNSA method, as described in reference [31]. A standardized protocol was employed to evaluate the *α*‐amylase inhibitory activity. The reaction mixture consisted of two hundred μL of phosphate buffer (Na_2_HPO_4_/NaH_2_PO_4_, 0.02 M, containing 0.006 M NaCl, pH 6.9), 100 μL of soluble starch solution (1% w/v in water) as the substrate, and 100 μL of diluted phenolic extract. The mixture was incubated at 37°C for 5 min. The enzymatic reaction was then initiated by adding one hundred μL of *α*‐amylase (EC 3.2.1.1) derived from *Aspergillus oryzae* (13 units). After a further incubation of 5 min at the same temperature, the reaction was terminated by the addition of 1 mL of DNSA reagent. The mixture was boiled for 5 min in a water bath at 100°C, cooled to room temperature, and diluted with 2 mL of distilled water. Absorbance was measured at 530 nm using a UV–visible spectrophotometer. A control representing 100% enzyme activity was prepared by replacing the plant extract with one hundred μL of buffer. A blank was also prepared for each extract concentration in the absence of the enzyme solution. Acarbose (50 mg, Gluconova) was used as the reference standard.

All measurements were performed in triplicate. The percentage inhibition was calculated using the same PI% equation as above.

The inhibitory capacity was also expressed as AACE (μmol acarbose equivalent per gram of extract), calculated using the following formula:

(4) AACEµmol/g=PIK∗F∗VP,

where PI: inhibition percentage, *K*: slope of the calibration curve, *F*: dilution factor, *V*: volume of solvent, and *p*: sample mass.

### 2.6. Statistical Analysis

Statistical analysis was conducted using two‐way analysis of variance (ANOVA) to evaluate the effects of both geographical origin and maturity stage, as well as their interaction, on the parameters studied. When significant differences were detected, a post‐hoc multiple comparison test (LSD or Tukey HSD, as appropriate) was applied to determine specific differences between group means. Letter designations (e.g., “a,” “b,” and “c”) presented in the tables indicate statistically homogeneous groups; values sharing the same letter are not significantly different (*p* > 0.05), while different letters denote significant differences (*p* < 0.05).

## 3. Results and Discussion

### 3.1. Oil Content

The oil extraction results from *Pistacia atlantica* fruits revealed a clear influence of both the ripening stage and geographical location on the extraction yield. It was observed that semiripe fruits (RF) exhibited higher oil yields compared to unripe fruits (RC) in both studied regions. In region B, the oil yield increased from 17.26% in unripe fruits (BRC) to 24.62% in semiripe fruits (BRF), while in region S, it rose from 15.35% (SRC) to 24.78% (SRF). This increase reflects the gradual accumulation of lipids during fruit maturation, which is consistent with previous findings [32, 33], indicating that ripening is a key factor in enhancing oil yield.

Notably, unripe fruits record oil yields exceeding 15% in both regions, which is considered high during the early stages of ripening. These values are close to the minimum threshold of 17% established by the Food and Agriculture Organization (FAO) for seeds to be classified as suitable raw material for industrial oil production. In fact, the oil content in unripe fruits reached this threshold in region B and approached it in region S. At the semiripe stage, oil yields significantly exceeded this benchmark, further supporting the potential of these fruits as a promising source for oil production [34].

Moreover, the comparison between the two regions revealed relative differences in yield, with region B showing a slight advantage at the early ripening stage, whereas values were identical at the semiripe stage. This suggests that the geographical effect is more pronounced during the initial phases of fruit development and tends to diminish gradually as ripening progresses. Therefore, selecting the optimal harvest time is a critical factor for maximizing oil productivity, while also considering the environmental characteristics of each location.

### 3.2. Unsaponifiable Fraction

The USM refers to the lipid components that do not undergo saponification during the process and remain unreactive with alkalis. This fraction contains bioactive compounds such as sterols, tocopherols, carotenoids, terpenic alcohols, and squalene [35]. It is insoluble in water but soluble in organic solvents. USM is particularly rich in policosanols, phytosterols, squalene, and fat‐soluble vitamins, which have demonstrated antihyperlipidemic and antiphotoaging properties [36]. Therefore, it serves as an important chemical marker reflecting the oil’s quality and biochemical composition [37].

The results revealed a clear variation in the yield of the USM depending on both the geographical origin and the ripening stage of the fruits. As shown in Table 1, unripe fruits from the SRC region exhibited the highest yield of USM (10.45%) compared to the semiripe fruits from the same region (SRF: 5.36%). In contrast, lower values were recorded in the BRC and BRF regions (5.94% and 4.07%, respectively). This decline in yield with advancing ripeness may be attributed to physiological changes associated with reduced metabolic activity, degradation of sensitive compounds such as tocopherols and carotenoids, and processes like autoxidation or enzymatic breakdown [38].

**Table 1:** Oil yield (%) and unsaponifiable matter yield (%) of *Pistacia atlantica* fruits at unripe and semiripe stages from two regions in Djelfa Province.

Localities	Extraction of oil yield (%)	Unsaponifiable matter yield (%)
BRC^∗^	17.26a	5.94b
SRC^∗∗^	15.35b	10.45a
LSDp0.05	0.95	0.48
BRF^∗∗∗^	24.62	4.07b
SRF^∗∗∗∗^	24.78	5.36a
LSDp0.05	ns^∗∗∗∗∗^	0.37

*Note:* Letter designations in tables, such as “a,” “b,” “c,” and “d” (e.g., 17.26a), refer to the results of a statistical analysis, usually a test of comparison of means (e.g., ANOVA test with a post‐hoc test such as LSD or Tukey HSD). They mean that values with the same letter are not statistically significantly different at a given level of significance (most often *p* < 0.05). Values with different letters are statistically significantly different at the same level of significance. BRC^∗^: Berrezigue Region–Crude/Unripe stage. SRC^∗∗^: Seddary Region–Crude/Unripe stage. BRF^∗∗∗^: Berrezigue Region–Full‐ripe/Semiripe stage. SRF^∗∗∗∗^: Seddary Region–Full‐ripe/Semiripe stage. LSDp0.05: Least Significant Difference p < 0.05. This is a threshold value–if the difference between two means is greater than the LSD, it is considered statistically significant.

^∗^Explain all the symbols in the table.

^∗∗∗∗∗^Not significant at p0.05.

The results related to oil extraction efficiency and USM content from *Pistacia atlantica* fruits collected from two different regions in the Djelfa Province (BRC/SRC and BRF/SRF) and at two different phenological stages (unripe and semiripe) demonstrated a significant influence of both geographical location and ripening stage. In the BRC/SRC region, a statistically significant difference was observed in oil yield, with unripe fruits (BRC) showing higher extraction efficiency, indicating a greater fat content compared to semiripe fruits (SRC). This unusual trend may reflect the influence of local environmental conditions. Regarding the USM, semiripe fruits (SRC) showed significantly higher content (10.45%) than unripe ones (5.94%), suggesting that the accumulation of bioactive compounds (such as sterols and tocopherols) increases with ripening.

In the second region (BRF/SRF), oil yields did not differ significantly between unripe (24.62%) and semiripe fruits (24.78%), indicating that ripening had no impact on oil content in this area. However, the content of USM was significantly higher in semiripe fruits (SRF: 5.36%) compared to unripe ones (BRF: 4.07%) with an LSD of 0.37, confirming a similar trend to that observed in the first region.

Collectively, these results suggest that oil yield may be region‐dependent: a visible decrease with ripening was observed in one region (BRC/SRC), while it remained stable in the other (BRF/SRF). On the other hand, the USM increased with ripening in both regions, indicating that semiripe fruits are richer in bioactive compounds. These regional differences may be due to environmental factors such as soil and climate or genetic variations affecting metabolic accumulation dynamics. These findings emphasize the importance of ripening stage and geographical location as key factors in guiding the targeted use of *Pistacia atlantica* fruits for high‐value nutritional and functional applications, particularly for the extraction of unsaponifiable compounds with proven biological properties.

#### 3.2.1. Total Tocopherols

Tocopherols, especially *α*‐tocopherol, are known as potent antioxidant compounds, responsible for protecting unsaturated fatty acids from oxidative damage, thereby contributing to the enhancement of oil stability [39].

The amount of tocopherols in the tested extracts was determined based on the linear standard curve equation of *α*‐tocopherol, plotted as a function of absorbance against a series of increasing concentrations (*y* = 21.145×, *r* ^2^ = 0.999). The values calculated from the equation were expressed in units of (mg *α*‐tocopherol equivalent/g of oil), as presented in Table 2.

**Table 2:** Concentrations of functional compounds in the crude oil and in the unsaponifiable fraction of selected localities of *Pistacia atlantica* fruit oil.

Localities	Concentrations of functional compounds in the unsaponifiable fraction			Concentrations of functional compounds in the crude oil		
	Total carotenoids (mg *β*CE/g USM)	Total sterols (mg *β*S/g USM)	Total tocopherols (mg *α*TE/g USM)	Carotenoids (mg *β*CE/g oil)	Sterols (mg *β*S/g oil)	Tocopherols (mg *α*TE/g oil)
BRC^∗^	19.48b^∗∗∗∗∗^ ± 0.18	847.04a ± 51.80	8.88b ± 0.09	1.16b	50.31b	0.53b
SRC^∗∗^	20.76a ± 1.46	871.36a ± 53.85	4.24c ± 0.09	2.17a	91.03a	0.44c
BRF^∗∗∗^	15.22c ± 1.11	844.45a ± 23.13	17.88a ± 0.98	0.62c	34.37 d	0.73a
SRF^∗∗∗∗^	20.62a ± 0.66	865.94 a± 25.46	3.10 d ± 0.09	1.11b	46.42cd	0.17 d
LSDp_0.05_	1.10	49.72	0.51	0.07	3.11	0.03

*Note:* Values with different letters are statistically significantly different at the same level of significance. BRC^∗^: Berrezigue Region–Crude/Unripe stage. SRC^∗∗^: Seddary Region–Crude/Unripe stage. BRF^∗∗∗^: Berrezigue Region–Full‐ripe/Semiripe stage. SRF^∗∗∗∗^: Seddary Region–Full‐ripe/Semiripe stage. LSDp0.05: Least Significant Difference p < 0.05. This is a threshold value–if the difference between two means is greater than the LSD, it is considered statistically significant.

^∗∗∗∗∗^Letters are not statistically significantly different at a given level of significance (most often *p* < 0.05).

As shown in Table 2, tocopherol levels exhibited the highest degree of variability among the analyzed bioactive compounds, both in the unsaponifiable fraction and in the crude oil, depending on geographical origin and ripening stage. In region B, the semiripe fruits (BRF) demonstrated the highest tocopherol concentration, reaching 17.88 ± 0.98 mg αTE/g USM, significantly surpassing that of the unripe fruits (BRC, 8.88 ± 0.09 mg/g), indicating a positive correlation between ripening and tocopherol accumulation in this region. Conversely, region S showed an inverse pattern, where the tocopherol content decreased from 4.24 ± 0.09 mg/g in unripe fruits (SRC) to 3.10 ± 0.09 mg/g in semiripe fruits (SRF), suggesting that ripening negatively affects tocopherol biosynthesis or stability in this environment.

A similar trend was observed in the crude oil: BRF recorded the highest tocopherol content (0.73 mg/g oil), followed by BRC (0.53 mg/g). In contrast, lower concentrations were measured in region S, with 0.44 mg/g in SRC and only 0.17 mg/g in SRF. These findings highlight that the oil derived from fully ripe fruits in region B represents a particularly rich source of tocopherols. Meanwhile, region S exhibited lower tocopherol levels across both maturity stages, due to environmental constraints affecting metabolic pathways related to tocopherol synthesis and preservation. This variability underscores the importance of considering both geographical and phenological factors in evaluating the nutritional and functional potential of Pistacia atlantica oils.

These findings indicate that tocopherol accumulation does not follow a uniform pattern with ripening but rather is influenced by the local environmental context. While concentrations increase with maturity in region B, they decline in region S, revealing a marked ecological variability in accumulation dynamics. When compared to the study by Guenane et al. [40], tocopherol content was higher in unripe fruits (50.7 mg/100 g oil) than in ripe ones (34.1 mg/100 g oil), a trend that partially aligns with the pattern observed in region S. Conversely, the findings of Labdelli et al. [41] reported an increase in tocopherols with ripening, consistent with the observation in BRF.

#### 3.2.2. Total Carotenoids

Carotenoids are biologically active lipid compounds characterized by their antioxidant activity, in addition to their role as natural pigments and precursors of vitamin A. They contribute to enhancing the oxidative stability of oil and endowing it with additional nutritional and functional properties [42].

The amount of carotenoids in the tested extracts was determined based on the linear standard curve equation of *β*‐carotene, plotted as a function of absorbance against a series of increasing concentrations (*y* = 18.367*x* + 0.0366, *R* ^2^ = 0.9986). The values calculated from the equation were expressed in units of (mg *β*‐carotene equivalent/g of oil), as presented in Table 2.

As presented in Table 2, the carotenoid content demonstrated significant variability between the two studied regions (S and B) and across different ripening stages. In the USM, the highest concentrations were recorded in region S, with values of 20.76 ± 1.46 mg *β*CE/g USM in SRC and 20.62 ± 0.66 mg *β*CE/g USM in SRF, showing no significant difference between the unripe and semiripe stages. Conversely, region B exhibited comparatively lower carotenoid levels, with 19.48 ± 0.18 mg/g in BRC, decreasing significantly to 15.22 ± 1.11 mg/g in BRF, reflecting a notable decline during ripening at this site.

A similar trend was observed in the crude oil, where SRC again displayed the highest carotenoid content (2.17 mg/g oil), followed by SRF (1.11 mg/g). In contrast, region B showed a marked reduction in carotenoid concentration from 1.16 mg/g in BRC to only 0.62 mg/g in BRF. These findings indicate that region S provides more favorable environmental conditions for carotenoid accumulation in both the unsaponifiable fraction and the crude oil, thereby enhancing the phytochemical value of the oil as a source of antioxidant and pigment‐active compounds. The significant decrease observed in region B, particularly at full ripeness, may suggest a lower carotenoid biosynthesis capacity or increased degradation due to environmental or physiological factors.

These findings emphasize that carotenoids are more abundant in unripe fruits, which may be attributed to their high sensitivity to oxidative, photolytic, and thermal factors accompanying ripening. However, the relative stability of carotenoid content in region S, even in ripe fruits, suggests the presence of local environmental factors that contribute to maintaining the stability or continued synthesis of these compounds. This interpretation is supported by literature reports on carotenoid degradation through oxidation or metabolic transformation during ripening [43, 44].

Compared to previous studies, notably Labdelli et al. [41], the values recorded in this study are significantly higher, especially after unit standardization—highlighting the exceptional bioactive potential of *Pistacia atlantica* fruits, particularly the unripe ones from Djelfa region, in terms of carotenoid richness. This superiority is partly attributed to the use of an analytical method based on unsaponifiable fraction extraction, which provides greater accuracy in estimating these compounds compared to conventional methods based on direct oil analysis.

These findings underscore that the actual estimation of carotenoids must consider not only their concentration in the USM but also the yield of this fraction, as high concentrations do not necessarily reflect the final content in the oil without efficient extraction. Moreover, the decline observed with ripening, especially in region B, highlights the importance of controlling environmental conditions and harvest timing to preserve the biochemical quality of the fruits.

#### 3.2.3. Total Sterols

Phytosterols are unsaponifiable compounds that play a vital role in the structure of plant cell membranes and are known for their health‐promoting effects, particularly in reducing cholesterol absorption [45]. They are stable constituents in vegetable oils and are used as markers of oil quality and refinement.

The amount of sterols in the tested extracts was determined based on the linear standard curve equation of *β*‐sitosterol, plotted as a function of absorbance against a series of increasing concentrations (*y* = 0.4276×, *r* ^2^ = 0.9983). The values calculated from the equation were expressed in units of mg *β*‐sitosterol equivalent/g of oil, as shown in Table 2.

The results presented in Table 2 revealed that sterol concentrations in the USM were consistently high across all samples, with slightly higher values recorded in region S compared to region B. In Sougaa, the unripe fruits (SRC) exhibited the highest content (871.36 ± 53.85 mg *β*S/g USM), followed closely by the semiripe fruits (SRF, 865.94 ± 25.46 mg/g USM), indicating minimal fluctuation during ripening. Similarly, in Bousdraya, sterol content was also high, albeit slightly lower, with values of 847.04 ± 51.80 mg/g in BRC and 844.45 ± 23.13 mg/g in BRF. Statistical analysis confirmed that all samples shared comparably elevated levels of sterols in the unsaponifiable fraction, with no significant differences between localities. In contrast, more pronounced variations were observed in the crude oil. SRC stood out with a markedly higher sterol concentration (91.03 mg *β*S/g oil), followed by SRF (46.42 mg/g), while BRC and BRF recorded lower values (50.31 and 34.37 mg/g, respectively). These discrepancies suggest that although sterols are concentrated in USM, their distribution in crude oil does not always mirror that of the unsaponifiable fraction. This highlights the critical role of USM yield in accurately assessing the oil’s functional compound content. Overall, the findings underscore the favorable conditions of region S, particularly at the unripe stage, for enhancing phytosterol accumulation—compounds known for their cholesterol‐lowering effects—while also pointing to a higher sensitivity to ripening and potential environmental modulation in region B.

Table 2 presents the concentrations of total carotenoids, total sterols, and total tocopherols in both the crude oil and the unsaponifiable fraction of *Pistacia atlantica* fruit oil from various localities (BRC, SRC, BRF, and SRF). The data reveal notable differences in compound concentrations across localities and between the crude oil and unsaponifiable fractions.

In conclusion, the results presented in Table 2 highlight the biological significance of the unsaponifiable fraction as a concentrated and rich source of functional bioactive compounds, including carotenoids, sterols, and tocopherols, in comparison to the crude oil. This difference is expected, as the USM naturally contains fat‐soluble vitamins and compounds such as carotenoids, explaining their higher concentrations in this fraction. The SRC locality stood out for its notably high content of carotenoids and sterols, while the BRF samples exhibited elevated levels of tocopherols. These variations reflect the influence of environmental and geographical factors on the phytochemical profile of *Pistacia atlantica* fruit oil. The statistical values (LSDp0.05) support the observed differences, confirming that they are significant and not due to random variation. These findings emphasize the potential of this oil as a valuable natural source of bioactive compounds and underline the importance of selecting the appropriate geographical origin and ripening stage to maximize its health benefits and industrial applications.

### 3.3. Biological Activity of Unsaponifiable Compounds

In addition to their chemical and nutritional importance, unsaponifiable compounds in vegetable oils are considered valuable bioactive sources with high biological relevance. They play a significant role in combating oxidative stress and regulating various enzymes associated with metabolic disorders.

In this context, the biological activity of the unsaponifiable fraction extracted from *Pistacia atlantica* fruits—collected from different geographical regions and ripening stages—was assessed using two key bioassays: the DPPH radical scavenging activity and the inhibitory effect on *α*‐amylase enzyme**.**

#### 3.3.1. Antioxidant Activity (DPPH Scavenging Activity)

The antioxidant activity was evaluated using the IC_50_ value, which represents the concentration required to inhibit 50% of the DPPH free radicals. Lower IC_50_ values indicate higher antioxidant potency. Table 3 shows the antioxidant activity of the nonsaponifiable fractions from *Pistacia atlantica* fruit oil, measured by the DPPH radical scavenging assay (IC_50_ values). The IC_50_ value is the sample concentration required to inhibit 50% of the DPPH free radicals. The lower the IC_50_ value, the stronger the antioxidant activity. Vitamin C serves as a reference standard.

**Table 3:** Antioxidant activity of unsaponifiable fractions from *Pistacia atlantica* fruit oil measured by DPPH radical scavenging assay (IC_50_ values).

Specification	DPPH (IC_50_, mg/mL)
BRC^∗^	1.27a^∗∗∗∗∗^ ± 0.13
SRC^∗∗^	0.97c ± 0.01
BRF^∗∗^	0.49 days ± 0.06
SRF^∗∗∗^	1.06b ± 0.05
LSDp0.05	0.06
Vitamin E (std.)	0.0195 ± 0.0075
Vitamin C (std.)	0.013 ± 0.005

*Note:* Values with different letters are statistically significantly different at the same level of significance.

^∗,∗∗,∗∗∗,∗∗∗∗∗^They mean that values with the same letter are not statistically significantly different at a given level of significance (most often *p* < 0.05).

The antioxidant potential of the unsaponifiable fractions from *Pistacia atlantica* fruit oil was evaluated using the DPPH radical scavenging assay, and the results are presented in Table 3. Significant differences (*p* < 0.05) in IC_50_ values were observed between regions and ripening stages, as confirmed by the LSD test (LSD_0_._05_ = 0.06).

The most potent antioxidant activity was recorded in the semiripe sample from the Bousdraya region (BRF), with an IC_50_ value of 0.49 ± 0.06 mg/mL, statistically distinct from all other tested samples. This finding underscores the high efficacy of the oil extract from this location and maturity stage. The highest antioxidant activity, expressed by the lowest IC_50 value, was demonstrated by the BRF fraction, for which the IC_50 was 0.49 ± 0.06 mg/mL. A lower IC_50 value indicates higher activity. This fraction was statistically significantly (letter d) more potent than the other fractions. However, vitamin C (IC_50 = 0.013 ± 0.005 mg/mL) and vitamin E (IC_5 = 0.0195 ± 0.0075 mg/mL)—as pure standards—demonstrated several dozen times higher activity than the best unsaponifiable fraction.

In contrast, the unripe sample from the same region (BRC) exhibited the weakest activity (IC_50_ = 1.27 ± 0.13 mg/mL), confirming a significant enhancement in antioxidant capacity with ripening at this site. This trend suggests the biosynthesis or accumulation of potent antioxidant compounds, particularly tocopherols, during later developmental stages.

On the other hand, in the Sougaa region, the antioxidant activity followed an inverse pattern. The unripe sample (SRC) demonstrated stronger activity (IC_50_ = 0.97 ± 0.01 mg/mL) than the semiripe one (SRF: IC_50_ = 1.06 ± 0.05 mg/mL), indicating a decline in antioxidant potential with fruit maturation. This may reflect oxidative degradation or reduced synthesis of active compounds such as carotenoids, which were found in higher concentrations in SRC.

When comparing geographical origins at the same phenological stage, differences were also evident. At the unripe stage, SRC (Sougaa) showed superior antioxidant activity compared to BRC (Bousdraya), while at the semiripe stage, BRF outperformed SRF. These results clearly illustrate that both ripening stage and environmental conditions (e.g., solar radiation, temperature, and soil composition) significantly affect the bioactivity of the oil.

As anticipated, vitamin C and vitamin E, used as positive controls, showed markedly higher antioxidant activity (IC_50_ = 0.013 ± 0.005 mg/mL and IC_50_ = 0.195 ± 0.0075 mg/mL, respectively), underscoring the comparatively moderate effectiveness of complex plant‐derived fractions relative to pure reference standards.

Overall, these findings demonstrate that the antioxidant activity of *Pistacia atlantica* USM is influenced by a multifactorial interaction between developmental stage and ecological conditions. The semiripe fruits from Bousdraya (BRF) appear to offer the highest bioactivity, supporting the notion that optimized harvest timing and location are critical for maximizing the functional quality of *Pistacia atlantica* oil in food and pharmaceutical applications.

#### 3.3.2. *α*‐Amylase Inhibition

α‐Amylase is a key therapeutic target in the management of type 2 diabetes, as its inhibition reduces the rate of complex carbohydrate hydrolysis, thereby regulating postprandial blood glucose levels [48]. In this study, the inhibitory activity of the samples was expressed as acarbose equivalent units, reported in μmol of acarbose per gram of USM (μmol acarbose/g insapo).

The *α*‐amylase inhibitory activity of the tested extracts was determined based on the linear standard curve equation of acarbose, plotted as a function of absorbance against a series of increasing concentrations (*y* = 3.5076×, *R* ^2^ = 0.9881).

Table 4 shows the *α*‐amylase enzyme inhibitory activity for nonsaponifiable fractions of *Pistacia atlantica* fruit oil from various locations. This activity is expressed in acarbose equivalents μmol AACE/g USM, which means that a higher value indicates a stronger inhibitory effect. The letters (“a” and “b”) next to the values indicate statistical differences between means, where the same letters indicate no statistically significant differences and different letters indicate their existence, at a significance level of *p* < 0.05.

**Table 4:** *α*‐Amylase inhibitory activity of unsaponifiable fractions from *Pistacia atlantica* fruit oil expressed in acarbose equivalent (μmol AACE/g USM).

Localities	(μmol acarbose/g insapo) AACE
BRC	32.75b ± 0.47
SRC	32.14b ± 0.31
BRF	33.56a ± 0.17
SRF	34.58a ± 1.33
LSDp0.05	1.86

*Note:* They mean that values with the same letter are not statistically significantly different at a given level of significance (most often *p* < 0.05). Values with different letters are statistically significantly different at the same level of significance.

The *α*‐amylase inhibitory capacity of the USM from Pistacia atlantica oil varied significantly across the different geographic origins. The highest activity was observed in the fractions obtained from the SRF (34.58 ± 1.33 μmol AACE/g USM) and BRF (33.56 ± 0.17 μmol AACE/g USM) sites. These values, assigned with the same statistical letter “a,” indicate a comparable and significantly higher inhibitory effect relative to the other locations.

Conversely, USM fractions from the BRC (32.75 ± 0.47 μmol AACE/g USM) and SRC (32.14 ± 0.31 μmol AACE/g USM) sites displayed lower inhibitory activities. Both were statistically similar to each other (letter “b”) but significantly different from SRF and BRF.

These findings confirm that the nonsaponifiable fraction of *Pistacia atlantica* oil exerts a measurable *α*‐amylase inhibitory effect, which is of potential interest for glycemic regulation, as *α*‐amylase plays a significant role in carbohydrate hydrolysis. Notably, the inhibitory pattern differs from that observed for antioxidant activity, where BRF and SRC had the highest performances. In contrast, for *α*‐amylase inhibition, BRF retained high activity while SRC ranked among the least active. This suggests that compounds other than the main antioxidant constituents (e.g., tocopherols, carotenoids, and sterols) might be responsible for *α*‐amylase inhibition. This hypothesis is supported by the Pearson correlation matrix, which revealed no significant correlation between the levels of these bioactive compounds and *α*‐amylase inhibitory potential.

### 3.4. Correlation of Antioxidant Activity and *α*‐Amylase Inhibition With the Content of the Unsaponifiable Fraction Components

Table 5 presents the Pearson correlation matrix (*r*), which quantifies the strength and direction of the linear relationship between the components of nonsaponifiable fractions (tocopherols, carotenoids, and sterols) and two biological activities: antioxidant activity (IC_50_) and *α*‐amylase inhibition (AACE) (Table 5).

**Table 5:** Pearson’s correlation matrix (*r*) between unsaponifiable fraction components and biological activities.

Specification	Tocopherols (mg/g USM)	Carotenoids (mg/g USM)	Sterols (mg/g USM)	Antioxidant activity (IC_50_, mg/mL)	α‐Amylase inhibition (AACE, mmol/g)
Tocopherols (mg/g USM)	1.00	−0.79	−0.94	−0.92	+0.01
Carotenoids (mg/g USM)		1.00	+0.97	−0.56	−0.15
Sterols (mg/g USM)		+0.97	1.00	−0.46	−0.07
Antioxidant activity (IC_50_, mg/mL)				1.00	−0.17
α‐Amylase inhibition (AACE, mmol/g USM)					1.00

The correlation analysis between the components of the unsaponifiable fraction and the biological activities of Pistacia atlantica fruit oil revealed significant variations that reflect the influence of chemical composition on bioactivity. A strong negative correlation was observed between tocopherol content and both carotenoids (*r* = −0.79) and sterols (*r* = −0.94), indicating that higher concentrations of tocopherols are associated with lower levels of the other two compounds. In contrast, carotenoids and sterols were found to be very strongly positively correlated (*r* = +0.97), suggesting their simultaneous accumulation in the oil. Regarding antioxidant activity, tocopherols showed a strong negative correlation with IC_50_ values (*r* = −0.92), confirming their central role in enhancing antioxidant capacity. Meanwhile, carotenoids (*r* = −0.56) and sterols (*r* = −0.46) exhibited moderate negative correlations, indicating a less pronounced contribution. As for *α*‐amylase inhibition, the correlations were very weak and statistically insignificant for tocopherols (*r* = +0.01), carotenoids (*r* = −0.15), and sterols (*r* = −0.07), suggesting that these compounds are not the main contributors to this activity. Instead, the inhibitory effect may be attributed to other constituents present in the unsaponifiable fraction or to synergistic interactions among its components. These findings underscore the physiological importance of tocopherols as potent antioxidants, whereas sterols and carotenoids contribute to a lesser extent, with no clear impact on *α*‐amylase inhibitory activity.

## 4. Conclusions

The paper has shown that the unripe fruits of *Pistacia atlantica* are rich source of bioactive unsaponifiable compounds, such as tocopherols, sterols, and carotenoids, which exhibited significant antioxidants and 8‐amylase inhibitory properties. The findings show that fruit development during the initial stages of fruit growth is an ideal harvesting season to obtain fruit to its full capacity in terms of yielding health‐promoting compounds. These observations are more specifically applicable when it comes to the valorization of *P. atlantica* oils as natural functional food ingredients with possible nutritional and metabolic health advantages.

The fact that the chemical composition and biological activity of the fruits vary with the geographical origin and maturity period highlights the significance of the environmental and the phenological aspects in establishing the bioactive potential of the fruits. Correlation studies have shown tocopherols to be the primary contributor to antioxidant activity, and the place of carotenoids in the inhibition of *α*‐amylase is also not much and needs further clarification.

In general, these results are supportive of the applicability of *P. atlantica* oils in food and pharmaceutical industry, especially natural antioxidants, and enzyme modulators. The scope of future studies should be on the molecular description of active components and full substantiation of biological activity by in vivo and clinical trials, which will eventually lead to their eventual incorporation into nutraceuticals, functional foods, and novel therapeutic formulations.

## Conflicts of Interest

The authors declare no conflicts of interest.

## Funding

No funding was received for this manuscript.

## Data Availability

The data that support the findings of this study are available from the corresponding author upon reasonable request.

## References

[1] Tian M. , Bai Y. , Tian H. , and Zhao X. , The Chemical Composition and health-promoting Benefits of Vegetable Oils—A Review, Molecules. (2023) 28, no. 17, 10.3390/molecules28176393. PMC10489903 37687222

[2] Orsavova J. , Misurcova L. , Vavra Ambrozova J. , Vicha R. , and Mlcek J. , Fatty Acids Composition of Vegetable Oils and Its Contribution to Dietary Energy Intake and Dependence of Cardiovascular Mortality on Dietary Intake of Fatty Acids, International Journal of Molecular Sciences. (2015) 16, no. 6, 12871–12890, 10.3390/ijms160612871, 2-s2.0-84931274980. 26057750 PMC4490476

[3] Tavakoli J. , Hajpour Soq K. , Yousefi A. , Estakhr P. , Dalvi M. , and Mousavi Khaneghah A. , Antioxidant Activity of *Pistacia atlantica* Var Mutica Kernel Oil and It Is Unsaponifiable Matters, J Food Sci Technol. (2019) 56, no. 12, 5336–5345, 10.1007/s13197-019-04004-0, 2-s2.0-85070634384. 31749481 PMC6838301

[4] Poljšak N. , Kreft S. , and Kočevar Glavač N. , Vegetable Butters and Oils in Skin Wound Healing: Scientific Evidence for New Opportunities in Dermatology, Phytotherapy Research. (2020) 34, no. 2, 254–269, 10.1002/ptr.6524. 31657094

[5] Kokalj Ladan M. and Kočevar Glavač N. , Statistical FT-IR Spectroscopy for the Characterization of 17 Vegetable Oils, Molecules. (2022) 27, no. 10, 10.3390/molecules27103190. PMC9147165 35630666

[6] Schoss K. and Glavač N. K. , Supercritical CO_2_ Extraction vs. Hexane Extraction and Cold Pressing: Comparative Analysis of Seed Oils from Six Plant Species, Plants. (2024) 13, no. 23, 10.3390/plants13233409. PMC11644741 39683202

[7] Jiang Q. , Natural Forms of Vitamin E: Metabolism, Antioxidant, and Anti-inflammatory Activities and Their Role in Disease Prevention and Therapy, Free Radical Biology and Medicine. (2014) 72, 76–90, 10.1016/j.freeradbiomed.2014.03.035, 2-s2.0-84899952359. 24704972 PMC4120831

[8] Cabral C. E. and Klein M. R. S. T. , Phytosterols in the Treatment of Hypercholesterolemia and Prevention of Cardiovascular Diseases, Arquivos Brasileiros de Cardiologia. (2017) 109, no. 5, 475–482, 10.5935/abc.20170158, 2-s2.0-85038414134. 29267628 PMC5729784

[9] Gebregziabher B. S. , Gebremeskel H. , Debesa B. et al., Carotenoids: Dietary Sources, Health Functions, Biofortification, Marketing Trend and Affecting Factors – a Review, Journal of Agriculture and Food Research. (2023) 14, 10.1016/j.jafr.2023.100834.

[10] Jimenez-Lopez C. , Carpena M. , Lourenço-Lopes C. et al., Bioactive Compounds and Quality of Extra Virgin Olive Oil, Foods. (2020) 9, no. 8, 10.3390/foods9081014. PMC7466243 32731481

[11] Bentireche F. , Guenane H. , and Yousfi M. , Fatty Acids, the Unsaponifiable Matter, and Polyphenols as Criteria to Distinguish *Pistacia atlantica* Unripe Fruit Oil, Journal of the American Oil Chemists′ Society. (2019) 96, no. 8, 903–910, 10.1002/aocs.12209, 2-s2.0-85070394125.

[12] Bailey L. H. , Manual of Cultivated Plants, 1985, New York.

[13] Body B. , Economie Forestière Nord.-Africaine, Tome 1, Milieu Physique et Humain, 1948, Paryż, Larose, France.

[14] El Zerey-Belaskri A. , Ribeiro T. , Alcaraz M. L. et al., Molecular Characterization of *Pistacia atlantica* Desf. subsp. *Atlantica* (*Anacardiaceae*) in Algeria: Genome Size Determination, Chromosome Count and Genetic Diversity Analysis Using SSR Markers, Scientific Horticulture. (2018) 227, 278–287, 10.1016/j.scienta.2017.09.016, 2-s2.0-85042189334.

[15] Browicz K. , Rychter B. , Characterization of Areas, Anacardiaceae, Chorology of Trees and Shrubs in Southwest Asia and Adjacent Regions, 1991, 6, Polish Scientific Publishers, Warszawa-Poznan, 5–8.

[16] Rowshan V. , Bahmanzadegan A. , and Tarakemeh A. , Volatile Compounds of *Pistacia atlantica* Desf. Galls and Leaves by Combi-PAL System Technique, Technical Journal of Engineering and Applied Sciences (TJEAS). (2013) 3, no. 9, 796–798.

[17] Dorehgirae A. and Pourabdollah E. , Comparison of the Chemical Profile of Oil Extracted from *Pistacia atlantica* Subspecies *Cabulica* with *Pistacia atlantica* Subspecies Mutica, Pakistan Journal of Food Sciences. (2015) 25, no. 1, 1–6.

[18] Mozaffarian V. , Dictionary of Iranian Plant Names: Latin-English-Persian, 1998, Farhang Publisher: Moeser, Tehran, Iran, https://www.mazdapublishers.com/book/dictionary-of-iranian-plant-names.

[19] Bozorgi M. , Memariani Z. , Mobli M. , Salehi Surmaghi M. H. , Shams-Ardekani M. R. , and Rahimi R. , Five Pistacia Species (*P. vera*, *P. atlantica*, *P. erebinthus*, *P. khinjuk*, and *P. lentiscus.*). A Review of Their Traditional Uses, Phytochemistry, and Pharmacology, The Scientific World Journal. (2013) 15, 10.1155/2013/219815, 2-s2.0-84893869142. PMC3876903 24453812

[20] Azizi F. , Gorji N. , Jokar R. , Rezghi M. , Shirafkan H. , and Moeini R. , The Effects of *Pistacia atlantica* Desf. fruit Oil on Primary Knee Osteoarthritis: a Randomized Controlled Clinical Trial, Journal of Ethnopharmacology. (2025) 342, 10.1016/j.jep.2025.119387. 39855435

[21] Zerkani H. , Amalich S. , Tagnaout I. , Bouharroud R. , and Zair T. , Chemical Composition, Pharmaceutical Potential, and Toxicity of the Essential Oils Extracted from the Leaves, Fruits, and Barks of *Pistacia atlantica* , Biocatalysis and Agricultural Biotechnology. (2022) 43, 10.1016/j.bcab.2022.102431.

[22] Labdelli A. , Foughalia A. , Tahirine M. et al., Lipid Content and Composition of *Pistacia atlantica* Desf. Subsp. *Atlantica* Fruits from Three Geographic Origins in Algeria, Vegetos. (2023) 36, no. 4, 1211–1219, 10.1007/s42535-022-00524-x.

[23] Bassouya M. , Chedadi M. , El fadili M. et al., Chemical, Mineralogical, and Biological Properties of *Pistacia atlantica* Subsp. *Atlantica* Essential Oils from the Middle Atlas of Morocco, Horticulturae. (2025) 11, no. 3, 10.3390/horticulturae11030265.

[24] Khammassi M. , Amato G. , Caputo L. et al., Fatty Acid Profiles and Biological Activities of the Vegetable Oils of *Argania spinosa, Pinus halepensis* and *Pistacia atlantica* Grown in Tunisia: a Preliminary Study, Molecules. (2024) 29, no. 1, 10.3390/molecules29010160. PMC10779628 38202742

[25] AOAC , Official Methods of Analysis, 2000, The Association of Official Analytical Chemists.

[26] Dib M. A. , Paolini J. , Bendahou M. , Varesi L. , Allali H. , Desjobert J. M. , Tabti B. , and Costa J. , Chemical Composition of Fatty Acid and Unsaponifiable Fractions of Leaves, Stems and Roots of Arbutus unedo and in Vitro Antimicrobial Activity of Unsaponifiable Extracts, Natural Product Communications. (2010) 5, no. 7, 10.1177/1934578X1000500721. 20734946

[27] Harkat H. , Bousba R. , Benincasa C. et al., Assessment of Biochemical Composition and Antioxidant Properties of Algerian Date Palm (*Phoenix dactylifera* L.) Seed Oil, Plants. (2022) 11, no. 3, 10.3390/plants11030381. PMC8838170 35161362

[28] Labhar A. , El-Mernissi Y. , Alla K. A. et al., Influence of the Seasons on the Chemical Composition and Biological Properties of *Pistacia lentiscus* L. Essential Oil in the Mediterranean Region, Scientific Reports. (2025) 15, no. 1, 10.1038/s41598-025-08833-2. PMC12350941 40804051

[29] Chelghoum M. , Guenane H. , Tahri D. et al., Influence of Altitude, Precipitation, and Temperature Factors on the Phytoconstituents, Antioxidant, and α-amylase Inhibitory Activities of *Pistacia atlantica* , Food Measure. (2021) 15, no. 5, 4411–4425, 10.1007/s11694-021-01006-5.

[30] Harrat M. , Benalia M. , Gourine N. , and Yousfi M. , Variability of the Chemical Compositions of Fatty Acids, Tocopherols and Lipids Antioxidant Activities, Obtained from the Leaves of *Pistacia lentiscus* L. Growing in Algeria, Mediterranean Journal of Nutrition and Metabolism. (2018) 11, no. 2, 1–17, 10.3233/MNM-18198, 2-s2.0-85049525937.

[31] Abirami A. , Nagarani G. , and Siddhuraju P. , *In Vitro* Antioxidant, Anti-Diabetic, Cholinesterase and Tyrosinase Inhibitory Potential of Fresh Juice from *Citrus Hystrix* and *C. maxima* Fruits, Food Science and Human Wellness. (2014) 3, no. 1, 16–25, 10.1016/j.fshw.2014.02.001.

[32] Rogiers S. Y. , Kumar G. M. , and Knowles N. R. , Maturation and Ripening of Fruit of *Amelanchier antifolic* Nutt. are Accompanied by Increasing Oxidative Stress, Publisher: Oxford University Press. (1998) 81, no. 2, 10.1006/anbo.1997.0543, 2-s2.0-0031889539.

[33] Sachan N. and Kumar V. , A Critical Review on Physiological Changes During Growth Maturation and Ripening of Citrus Fruits, European Journal of Nutrition & Food Safety. (2022) 14, no. 11, 146–159, 10.9734/ejnfs/2022/v14i111272.

[34] Akinoso R. and Raji A. O. , Optimization of Oil Extraction from Locust Beans Using Response Surface Methodology, Lipid Sci. Technol.(2010) 113, no. 2, 245–252, 10.1002/ejlt.201000354, 2-s2.0-79551690955.

[35] Czaplicki S. , Ogrodowska D. , Derewiaka D. , Tańska M. , and Zadernowski R. , Bioactive Compounds in Unsaponifiable Fraction of Oils from Unconventional Sources, European Journal of Lipid Science and Technology. (2011) 113, no. 12, 1456–1464, 10.1002/ejlt.201000410, 2-s2.0-83655182880.

[36] An M. , Heo H. , Park J. , Jeong H.-S. , Kim Y. , and Lee J. , Unsaponifiable Matter from Wheat Bran Cultivated in Korea Inhibits Hepatic Lipogenesis by Activating AMPK Pathway, Foods. (2023) 12, no. 21, 10.3390/foods12214016. PMC10650137 37959135

[37] Gharby S. and Charrouf Z. , Argan Oil: Chemical Composition, Extraction Process, and Quality Control, Frontiers in Nutrition. (2022) 8, 10.3389/fnut.2021.804587. PMC8850956 35187023

[38] Rosalie R. , Joas J. , Deytieux-Belleau C. et al., Antioxidant and Enzymatic Responses to Oxidative Stress are Induced by Pre-harvest Water Supply Reduction and Ripening on Mango (*Mangifera indica* L. Cv. Cog Shall’) in Relation to Carotenoid Content, Journal of Plant Physiology. (2015) 184, 68–78, 10.1016/j.jplph.2015.05.019, 2-s2.0-84938079300. 26232564

[39] Suárez-Jiménez G. M. , López-Saiz C. M. , Ramírez-Guerra H. E. , Ezquerra-Brauer J. M. , Ruiz-Cruz S. i , and Torres-Arreola W. , Role of Endogenous and Exogenous Tocopherols in the Lipid Stability of Marine Oil Systems: a Review, International Journal of Molecular Sciences. (2016) 17, no. 12, 10.3390/ijms17121968, 2-s2.0-84997419048. PMC5187768 27886145

[40] Guenane H. , Bentireche F. , Bellakhdar A. , Elhadj M. D. O. , and Yousfi M. , Total Tocopherol Content and Antioxidant Activity of Fruit Oil from *Pistacia atlantica* Desf. Growing Wild in Algeria, Der Pharma Chemica. (2017) 9, no. 13, 153–157, http://www.derpharmachemica.com/archive.html.

[41] Labdelli A. , Rebiai A. , Tahirine M. , Adda A. , and Merah O. , Nutritional Content and Antioxidant Capacity of the Seed and the Epicarp in Different Ecotypes of *Pistacia atlantica* Desf, Subsp. atlantica. Plants. (2020) 9, no. 9, 10.3390/plants9091065. PMC7570190 32825183

[42] Adamantidi T. , Lafara M.-P. , Venetikidou M. , Likartsi E. , Toganidou I. , and Tsoupras A. , Utilization and Bio-Efficacy of Carotenoids, Vitamin A, and Its Avitaminoses in Nutricosmetics, Cosmeceuticals, and Cosmetics’ Applications with Skin-Health Promoting Properties, Applied Sciences. (2025) 15, no. 3, 10.3390/app15031657.

[43] Mercadante A. Z. and Rodriguez-Amaya D. B. , Effects of Ripening, Cultivar Differences, and Processing on the Carotenoid Composition of Mango, Journal of Agricultural and Food Chemistry. (1998) 46, no. 1, 128–130, 10.1021/jf9702860, 2-s2.0-0000265188. 10554207

[44] Fambrini M. and Pugliesi C. , Carotenoids in Crops: Roles, Regulation of the Pathway, Breeding to Improve the Content, Beta Carotene Dietary Sources, Cancer, and Cognition, 2009.

[45] Fambrini M. and Puglesi C. , Carotenoids in Crops: Roles, Regulation of the Pathway, Breeding to Improve the Content, Book: Beta Carotene Dietary Sources, Cancer, and Cognition, 2009, Hauppauge, NY, USA, 1–57, 10.13140/2.1.2624.5927.

[46] Piironen V. , Lindsay D. G. , Miettinen T. A. , Toivo J. , and Lampi A. M. , Plant Sterols: Biosynthesis, Biological Function, and Their Importance to Human Nutrition, Journal of the Science of Food and Agriculture. (2000) 80, no. 7, 939–966.

[47] Li X. , Bai Y. , Jin Z. , and Svensson B. , Food-Derived Non-phenolic α-amylase and α-glucosidase Inhibitors for Controlling Starch Digestion Rate and Guiding diabetes-friendly Recipes, Let. (2022) 153, 10.1016/j.lwt.2021.112455.

[48] Fiorito S. , Preziuso F. , Sharifi-Rad M. , Marchetti L. , Epifano F. , and Genovese S. , Auraptene and Umbelliprenin: a Review on Their Latest Literature Acquisitions, Phytochemistry Reviews. (2022) 21, no. 2, 317–326, 10.1007/s11101-020-09713-5.

[49] Benmohamed M. , Guenane H. , Messaoudi M. et al., Mineral Profile, Antioxidant, Anti-Inflammatory, Antibacterial, Anti-Urease and Anti-α-Amylase Activities of the Unripe Fruit Extracts of *Pistacia atlantica* , Molecules. (2023) 28, no. 1, 10.3390/molecules28010349. PMC9824078 36615545

[50] Sharifi-Rad M. , Mohanta Y. K. , Pohl P. , Jaradat N. , Aboul-Soud M. A. , and Zengin G. , Variation of Phytochemical Constituents, Antioxidants, Antibacterial, Antifungal, and Anti-inflammatory Properties of *Grantia aucheri* (Boiss.) at Different Growth Stages, Microbial Pathogenesis. (2022) 172, 10.1016/j.micpath.2022.105805. 36179974

[51] Zengin G. , Uba A. I. , Ocal M. et al., Integration of in Vitro and in Silico Approaches to Assess Three Astragalus Species from Türkiye Flora: a Novel Spotlight from Lab Bench to Functional Applications, Food Bioscience. (2022) 49, 10.1016/j.fbio.2022.101858.

[52] Sharifi-Rad M. , Panda J. , Mohanta Y. K. , Pohl P. , Zengin G. , and Moloney M. G. , Essential Oil of *Cleome coluteoides* (Boiss.): Phytochemical Constituents, Antioxidant, Antimicrobial, Antiproliferative, Anti-inflammatory, Enzymatic Inhibition, and Xanthine Oxidase Inhibitory Properties, Journal of Herbal Medicine. (2025) 52, 10.1016/j.hermed.2025.101036.

